# Mib2 Regulates Lipid Metabolism in Heart Failure With Preserved Ejection Fraction via the Runx2–Hmgcs2 Axis

**DOI:** 10.1111/jcmm.70514

**Published:** 2025-03-30

**Authors:** Zhulan Cai, Shunyao Xu, Xiaohua Xiao, Chen Liu, Lingyun Zu

**Affiliations:** ^1^ Department of Cardiology and Institute of Vascular Medicine Peking University Third Hospital Beijing China; ^2^ State Key Laboratory of Vascular Homeostasis and Remodeling Peking University Beijing China; ^3^ NHC Key Laboratory of Cardiovascular Molecular Biology and Regulatory Peptides Peking University Beijing China; ^4^ Beijing Key Laboratory of Cardiovascular Receptors Research Beijing China; ^5^ Department of Critical Care Medicine Shenzhen People's Hospital, Second Clinical Medical College of Jinan University, First Affiliated Hospital of Southern University of Science and Technology Shenzhen China; ^6^ Department of Geriatrics The First Affiliated Hospital of Shenzhen University, Shenzhen Second People's Hospital Shenzhen China

**Keywords:** bioinformatics analysis, fatty acid metabolism, heart failure with preserved ejection fraction, Hmgcs2, Mib2, Runx2

## Abstract

Obesity and the mismanagement of lipids significantly contribute to the development of heart failure with preserved ejection fraction (HFpEF). However, the underlying molecular mechanisms that regulate the metabolic changes and disruptions in lipid balance within HFpEF remain to be fully understood. Transcriptome data for HFpEF were sourced from the National Center for Biotechnology Information (NCBI) database. A mouse model for HFpEF was developed utilising leptin‐deficient (ob/ob) mice. The cardiac‐specific mind bomb E3 ubiquitin protein ligase 2 (Mib2) overexpression in ob/ob mice was achieved by tail vein injection of a recombinant adeno‐associated virus serotype 9 vector carrying Mib2 with a cTNT promoter (AAV9‐cTNT‐Mib2). In vitro, neonatal rat ventricular myocytes were exposed to fatty acid to induce lipotoxicity. The molecular mechanisms were investigated through proteomic analysis, dual luciferase reporter gene assay, and immunoprecipitation assays. GO and KEGG enrichment analyses indicated that the differentially expressed proteins (DEPs) in HFpEF were prominently enriched in pathways related to the fatty acid metabolic process. The transcriptomic and proteomic analyses of heart tissues from HFpEF mice presented a notable elevation in the expression of 3‐hydroxy‐3‐methylglutaryl‐CoA synthase 2 (Hmgcs2). Immunoprecipitation assays revealed that mind bomb 2 (Mib2) directly interacted with runt‐related transcription factor 2 (Runx2), ubiquitinating and degrading Runx2 to inhibit Hmgcs2 transcription, impeding the fatty acid metabolic process. Mice with cardiac‐specific overexpression of Mib2 displayed a more pronounced progression of cardiac dysfunction and an accumulation of lipids compared to the control group. Our research uncovers a mechanism by which Mib2 modulates cardiac lipid metabolic homeostasis in HFpEF, implicating the Runx2‐Hmgcs2 axis.

**Abbreviations**
BP: biological process
CC: cellular component
DEGs: differentially expressed genes
DEPs: differentially expressed proteins
GO: gene ontology
GSEA: Gene Set Enrichment Analysis
HF: heart failure
HFpEF: heart failure with preserved ejection fraction
Hmgcs2: 3‐hydroxy‐3‐methylglutaryl‐CoA synthase 2
HW/TL: heart weight/tibia length
KEGG: Kyoto Encyclopedia of Genes and Genomes
LVEF: left ventricle ejection fraction
LVFS: left ventricle fractional shortening
LVIDd: left ventricle internal end‐diastolic diameter
LVIDs: left ventricle internal end‐systolic diameter
Mib2: mind bomb 2
NCBI: National Center for Biotechnology Information
NRVMs: neonatal rat ventricular myocytes
Runx2: runt‐related transcription factor 2

## 1 Introduction

Epidemiological evidence indicates that heart failure with preserved ejection fraction (HFpEF) is increasingly becoming the predominant form of heart failure (HF) globally [1, 2]. The Karolinska‐Rennes (KaRen) study disclosed that the 5‐year mortality rate for HFpEF stands at an alarming 47% [3]. After hospitalisation for HF, the 5‐year mortality rate for HFpE is approximately 65% [4]. Although this is an important medical issue, there are still limited effective therapeutic options available.

HFpEF is widely regarded as one of the most formidable challenges in cardiovascular diseases. Despite significant advancements in medical research, the development of effective therapeutic interventions for this syndrome has been remarkably slow. This dilemma is largely attributable to two critical factors. First, the biological mechanisms underlying the systemic pathophysiology of HFpEF remain incompletely understood [5, 6]. Second, HFpEF is characterised by substantial clinical phenotypic heterogeneity [7]. Such heterogeneity not only complicates diagnostic processes but also limits the efficacy of conventional ‘one‐size‐fits‐all’ therapeutic approaches. Given the pronounced variability in clinical phenotypes associated with HFpEF, traditional symptom‐focused research paradigms may fail to adequately capture the complexity of its pathophysiological mechanisms. Consequently, an aetiology‐driven research framework may offer greater scientific and clinical value. We aim to investigate the pathological mechanisms underlying HFpEF driven by metabolic disorders.

In recent years, with the significant increase in the incidence of multiple metabolic disorders such as obesity, diabetes and hypertension [1, 8, 9], HFpEF associated with metabolic syndrome, namely cardiometabolic HFpEF [10], has gradually become the most common form of HFpEF. The accumulation of body fat and its accompanying metabolic outcomes exert far‐reaching and protean effects both systemically and on the cardiovascular system [11]. This condition is characterised by the correlation between visceral fat and increased inflammation, insulin resistance, left ventricular hypertrophy, compromised left ventricular diastolic and systolic function, as well as issues with arterial, skeletal muscle and physical dysfunction, ultimately leading to symptomatic HFpEFPEVuZE5vdGU [12]. Most importantly, obesity‐associated metabolic syndrome frequently results in dysregulation of cardiac lipid metabolism, potentially contributing to diastolic dysfunction of HFpEF [13, 14]. Although there is a widely acknowledged link between lipid metabolic disorders and HFpEF, the specific mechanisms that lead to the accumulation of excess lipids in cardiomyocytes, referred to as cardiac lipid overload, remain inadequately understood. The primary aim of this study is to elucidate the key molecular pathways responsible for cardiac metabolic dysregulation and impaired lipid homeostasis in HFpEF driven by metabolic disorders.

In this study, we retrieved a collection of transcriptomic datasets derived from various modelling methods for cardiometabolic HFpEF, with the aim of identifying and comparing common differentially expressed genes (DEGs). Subsequently, we selected ob/ob mice as the cardiometabolic HFpEF model and further validated the protein expression levels of these DEGs using proteomics and western blotting. The choice of ob/ob mice as the HFpEF model was based on their pronounced metabolic disorder characteristics. Additionally, this cardiometabolic HFpEF model differs from the one used in the transcriptomic data, which further eliminates potential biases arising from model‐specific effects, thereby enhancing the generalisability and reliability of the findings.

Our findings indicate that cardiac metabolic alterations in HFpEF are associated with an upregulation of 3‐hydroxy‐3‐methylglutaryl‐CoA synthase 2 (Hmgcs2), a rate‐limiting enzyme in ketogenesis that involves in cell metabolism. Additionally, mind bomb 2 (Mib2) is implicated in triggering the ubiquitination and proteasomal degradation of runt‐related transcription factor 2 (Runx2), which in turn inhibits the transcription of Hmgcs2. The findings highlight the Mib2‐Runx2‐Hmgcs2 axis as a pivotal mechanism underlying the development of lipid abnormalities in cardiomyocytes.

## 2 Materials and Methods

### 2.1 Transcriptomic Datasets Retrieval

We performed a search and retrieved mRNA expression data for HFpEF from the Gene Expression Omnibus (GEO) database using the query ‘heart failure with preserved ejection fraction.’ Consequently, we chose and obtained datasets GSE163665, GSE240171, GSE235934, GSE249409, GSE218733 and GSE249411 for DEG analysis. All the aforementioned raw datasets are publicly accessible and can be downloaded from the GEO database. The platform annotation files associated with these GEO series (GSE) are detailed in Table 1.

**TABLE 1:** GEO datasets used in this study.

GEO accession	Platform ID	Model	Number of sample	Species	PMID
GSE163665	GPL18694 Illumina HiSeq 2500	ZSF1/ZSF1 obese rats	4 vs. 4	rat	33568522
GSE240171	GPL30172 NextSeq 2000	Control Vs AngII/HFD	3 vs. 3	mice	38363584
GSE235934	GPL23479 BGISEQ‐500	*wt* vs. *db/db*	2 vs. 3	mice	38957358
GSE249409	GPL24247 Illumina NovaSeq 6000	Control Vs HFD/mTAC	5 vs. 5	mice	38409164
GSE218733	GPL19057Illumina NextSeq 500	Control vs. HFD/L‐NAME	3 vs. 3	mice	39673349
GSE249411	GPL24247 Illumina NovaSeq 6000	Control Vs HFD/L‐NAME	4 vs. 4	mice	38409164

Abbreviations: AngII, angiotensin II; HFD, High‐Fat Diet; L‐NAME, N^ω^‐nitro‐l‐arginine methyl ester; mTAC, moderate transverse aortic constriction.

### 2.2 Identification of Differentially Expressed Genes

The ‘Limma’ package was utilised to identify DEGs between the HFpEF and the control group. The threshold for identifying DEGs was set with |log2 (fold change)| > 1 and *p*‐value < 0.05. The results of DEGs were visualised by Volcano Plots, which were created using the R package ‘ggplot2’.

### 2.3 Animal Study

C57BL/6J ob/ob male mice and wild‐type littermate controls were obtained from GemPharmatech Co. Ltd. (Jiangsu, China). To achieve cardiac‐specific overexpression of Mib2, adeno‐associated virus serotype 9 carrying Mib2 with cardiac troponin T (cTnT) promoter (AAV9‐cTnT‐Mib2) (Hanbio, China) was administered via tail vein injection to both ob/ob and wt mice. The efficiency of AAV‐mediated Mib2 gene overexpression was assessed 2 weeks post‐injection. The animals were maintained in an AAALAC‐accredited animal facility at Shenzhen University. All mouse‐related procedures were carried out in accordance with protocols approved by the Institutional Animal Care and Use Committee (IACUC) of Shenzhen University, adhering to the guidelines for the care and use of laboratory animals established by the National Academy of Sciences/National Research Council.

### 2.4 Echocardiography

Echocardiographic assessments were performed utilising a Small Animal Ultrasound Imaging System (Vevo2100, Canada) with 1% isoflurane. M‐mode and B‐mode assessments of the mid‐ventricular region were captured from the parasternal short‐axis perspective. The internal end‐diastolic diameter of the left ventricle (LVIDd) was recorded at the moment of the most pronounced diastolic dimension of the left ventricle, while the internal end‐systolic diameter (LVIDs) was captured at the time of the peak anterior movement of the posterior wall during systole. The left ventricular ejection fraction (LVEF) was determined using the formula for a cube: LVEF(%) = (LVIDd^3^−LVIDs^3^)/LVIDd^3^*100, and left ventricle fractional shortening (LVFS) was calculated with the formula: LVFS(%) = (LVIDd−LVIDs)/LVIDd*100. Measurements indicative of diastolic function were derived from the apical four‐chamber view, employing pulsed‐wave and tissue Doppler imaging.

### 2.5 Histopathological Examination

Pathological staining employed commercial kits, and the procedure was conducted according to the instructions provided by the manufacturer. For haematoxylin–eosin (HE) staining (Beyotime, Cat.C0105S), heart sections were incubated in a haematoxylin stain and subsequently soaked in eosin stain. For Masson's trichrome stain (Beyotime, Cat. C0189S), heart sections were stained with haematoxylin for 5 min, Azure B‐acid fuchsin for 10 min, and safranin for 1 min.

### 2.6 Random Blood Glucose Levels and Glucose Tolerance Tests and Insulin Tolerance Tests

Random blood glucose levels were investigated at 23 weeks. Mice at 23 to 24 weeks old underwent an intraperitoneal glucose tolerance test (ipGTT) with a dosage of 2 g/kg and an intraperitoneal insulin tolerance test (ipITT) with a dosage of 0.5 U/kg. Blood glucose levels were assessed at the initial baseline (0 min) and then at the following time points after injection: 15, 30, 60, 90 and 120 min using a glucometer (Roche Diagnostics, USA) and the area under the curve (AUC) was computed.

### 2.7 Biochemical Tests

The serum insulin concentration was measured using the Mouse Insulin ELISA Kit (Solarbio, Cat.SEKM‐0141) according to the manufacturer's instructions. The serum levels of ALT (alanine aminotransferase) and AST (aspartate aminotransferase), TG (triglyceride), CHO (cholesterol), LDL‐c (Low density lipoprotein cholesterol), HDL‐c (High density lipoprotein cholesterol), NEFA (nonestesterified fatty acid), CRP (C‐reactive protein) and LDH (lactate dehydrogenase) were detected using a Roche c311 automated biochemical analyser.

### 2.8 Proteomic Analysis

Protein extraction, quality evaluation and data‐independent acquisition (DIA) quantitative proteomic analysis were conducted at OEbiotech Corporation (Shanghai, China). The differentially expressed proteins (DEPs) were identified with a P‐value threshold of less than 0.05 and a fold change greater than 1.2. GO annotation, KEGG pathway annotation and Wiki Pathways enrichment analysis were conducted on the DEPs to identify terms that were significantly enriched. Gene Set Enrichment Analysis (GSEA) was conducted using the ‘cluster Profiler’ R package. The results were visualised using ‘ggplot2’ R package.

### 2.9 Western Blots

Protein samples were extracted from either left ventricular tissue or cultured cells utilising RIPA buffer (Solarbio, Cat.R0010) supplemented with protease and phosphatase inhibitors. The lysates were then centrifuged at 13,000 rpm for 10 min, and the supernatants thus obtained were employed for protein concentration measurement via the BCA Protein Assay Kit (Thermo Scientific, Cat. 23,225). Subsequently, equivalent quantities of protein extracts were separated by 10% SDS‐PAGE and transferred onto PVDF membranes. The membranes were pre‐incubated in 5% skim milk for 1 h, then incubated with primary antibodies overnight at 4°C, followed by incubation with secondary antibodies for 1 h at 37°C. The detection of protein signals was accomplished using an ECL chemiluminescent detection system (Yeasen Biotechnology, Cat. 36208ES60).

### 2.10 Plasmids and Transfection

The full‐length Mib2 and Runx2 plasmids were acquired by cloning their cDNA into the Phage vector with corresponding Flag or HA tag. The coding sequence of Runx2 was amplified from cDNA library and inserted into pcDNA3.1(+) expression vector between the NheI and BamH1 restriction sites. DNA fragments corresponding to the Hmgcs2 promoter region were amplified from cDNA library by PCR and inserted into pGL4.10 expression vector between the KPNI and XhoI restriction sites. HEK293 cells were maintained in Dulbecco's modified Eagle's medium (DMEM, Solarbio, Cat.11965) supplemented with 10% fetal bovine serum (FBS, Thermo Fisher, Cat.10099‐141C). For plasmid transfection, Lipofectamine 3000 (Invitrogen, Cat.L3000015) was utilised following the manufacturer's protocol.

### 2.11 Dual Luciferase Reporter Gene Assay

HEK293T cells were seeded in 24‐well plates and co‐transfected with firefly luciferase reporter plasmids encoding the Hmgcs2 promoter and pcDNA3.1 encoding Runx2. After 48 h of transfection, the luciferase activity of cell lysates was measured utilising a dual luciferase reporter gene assay system (Yeasen Biotechnology, Cat.11402ES60) by a multi‐mode microplate reader. Firefly activity was normalised to renilla luciferase activity.

### 2.12 Total RNA Extract and Quantitative Real‐Time PCR

Total mRNA was extracted from tissue and cultured neonatal rat ventricular myocytes (NRVMs) using the TRIzol reagent (Invitrogen, Cat.15596026). The extracted mRNA was quantified and subsequently reverse transcribed into cDNA using the Transcriptor First Strand cDNA Synthesis Kit (Roche, Cat.04379012001). Quantitative real‐time PCR (qPCR) was conducted using the SYBR Green PCR Master Mix (Vazyme, Cat.Q321). GAPDH served as the internal reference, and the 2‐ΔΔCT method was employed for the quantitative analysis of gene expression. The primer sequences used in the experiment are provided below.

The sequences of the primers.

**Table:** 

Gene	Species	Forward primer	Reverse primer
*GAPDH*	Mouse	ATGTGTCCGTCGTGGATCTG	AGTTGGGATAGGGCCTCTCTT
*Runx2*	Mouse	CCACCTCTGACTTCTGCCTC	ACTGGCGGGGTGTAGGTAAA
*GAPDH*	Rat	ACTCTACCCACGGCAAGTTC	TGGGTTTCCCGTTGATGACC
*Runx2*	Rat	CAGACACAATCCTCCCCACC	GCCAGAGGCAGAAGTCAGAG
*Hmgcs2*	Rat	CCCCGGGGAAAGAATGTGAG	GATCCTATGGGGTCGCTGTGTCT

### 2.13 Co‐Immunoprecipitation (Co‐IP) Assays

Heart tissues or HEK293T cells were lysed using cold immunoprecipitation (IP) buffer supplemented with a protease inhibitor cocktail and the phosphatase inhibitor PhosStop. The samples were fully lysed on ice, followed by centrifugation at 12,000 g for 10 min. Subsequently, 500 μL supernatants were incubated at 4°C overnight with 20 μL protein A + G magnetic beads (Beyotime, Cat.P2179S) and 1 μg corresponding antibody. After incubation, the beads were rinsed with cold IP buffer three times. Finally, the beads were heated with SDS‐PAGE sample loading buffer in preparation for subsequent Western blot analysis.

### 2.14 Ubiquitination Assay

HEK293T cells were co‐transfected with indicated His‐linked (His‐ubiquitin), HA‐Runx2 and Flag‐Mib2 plasmids, respectively. Then, they were lysed with lysis buffer including protease inhibitor cocktail. The lysates were centrifuged at 12,000 rpm for 10 min at 4°C, and the supernatants were subjected to IP assays with BeyoMag Anti‐HA magnetic beads (Beyotime, Cat.P2185S) at 4°C overnight. The beads from the immunoprecipitation process were thoroughly rinsed with a lysis buffer, followed by elution using an SDS‐containing sample buffer, and subsequently examined through Western blots. Ubiquitinated Runx2 was detected with anti‐His antibody.

### 2.15 Primary Cardiomyocytes Isolation and Cell Treatment

Hearts from neonatal Sprague–Dawley rats, aged 0.5 days, were subjected to dissociation and enzymatic digestion using trypsin and type II collagenase. Once the fibroblasts were eliminated, NRVMs were plated in culture dishes and maintained in DMEM that was supplemented with 10%FBS, 0.2 mM 5‐bromo‐2‐deoxyuridine and 1% penicillin/streptomycin at 37°C with 5% CO_2_. The NRVMs were treated with a mixture of fatty acids (FA), comprising a 1:1 ratio of palmitate (PA) to oleate (OA), at a concentration of 200 μM for 24 h.

### 2.16 Adenovirus and siRNA Transduction

Adenovirus encoding Mib2(ad‐Mib2) and their corresponding control vectors(ad‐NC) were constructed and purchased from HanBio Co. Ltd. (Shanghai, China). NRVMs were infected with ad‐Mib2 at a multiplicity of infection (MOI) of 50. Control small siRNA oligos, as well as siRNAs specifically targeting genes including *Mib2* and *hmgcs2* were procured from HanBio Co. Ltd. (Shanghai, China). NRVMs were transfected with siRNA using Lipofectamine RNAiMAX (Thermo Fisher, Cat.13778150) transfection reagent following the manufacturer's instructions.

### 2.17 Measurements of ATP Levels

Cellular ATP levels were determined in NRVMs by an ATP Assay Kit (Beyotime, Cat.S0027) following the manufacturer's protocol. Briefly, the cells were lysed, centrifuged, and the supernatant was used to measure intracellular ATP. A detecting solution was incubated at room temperature for 5 min in a 96‐well plate, to which the supernatants were added and mixed before luminescence signals were read by a microplate reader.

### 2.18 Oil O Staining

Cells were washed with PBS and fixed with formaldehyde for 10 min, then stained with Oil Red O (Beyotime, Cat.C0158S) for 20 min. Finally, the cells were re‐stained with haematoxylin for nuclear staining, and images were captured under a microscope.

### 2.19 Statistical Analysis

Data are presented as mean ± SD. Single‐factor comparison between the two groups was performed by *t*‐test. The comparison between two factors and multiple groups was conducted using a two‐way ANOVA analysis of variance. *p* < 0.05 was considered to be significant.

## 3 Results

### 3.1 Hmgcs2 Is a Commonly Differentially Expressed Gene in Metabolic HFpEF

**FIGURE 1:**
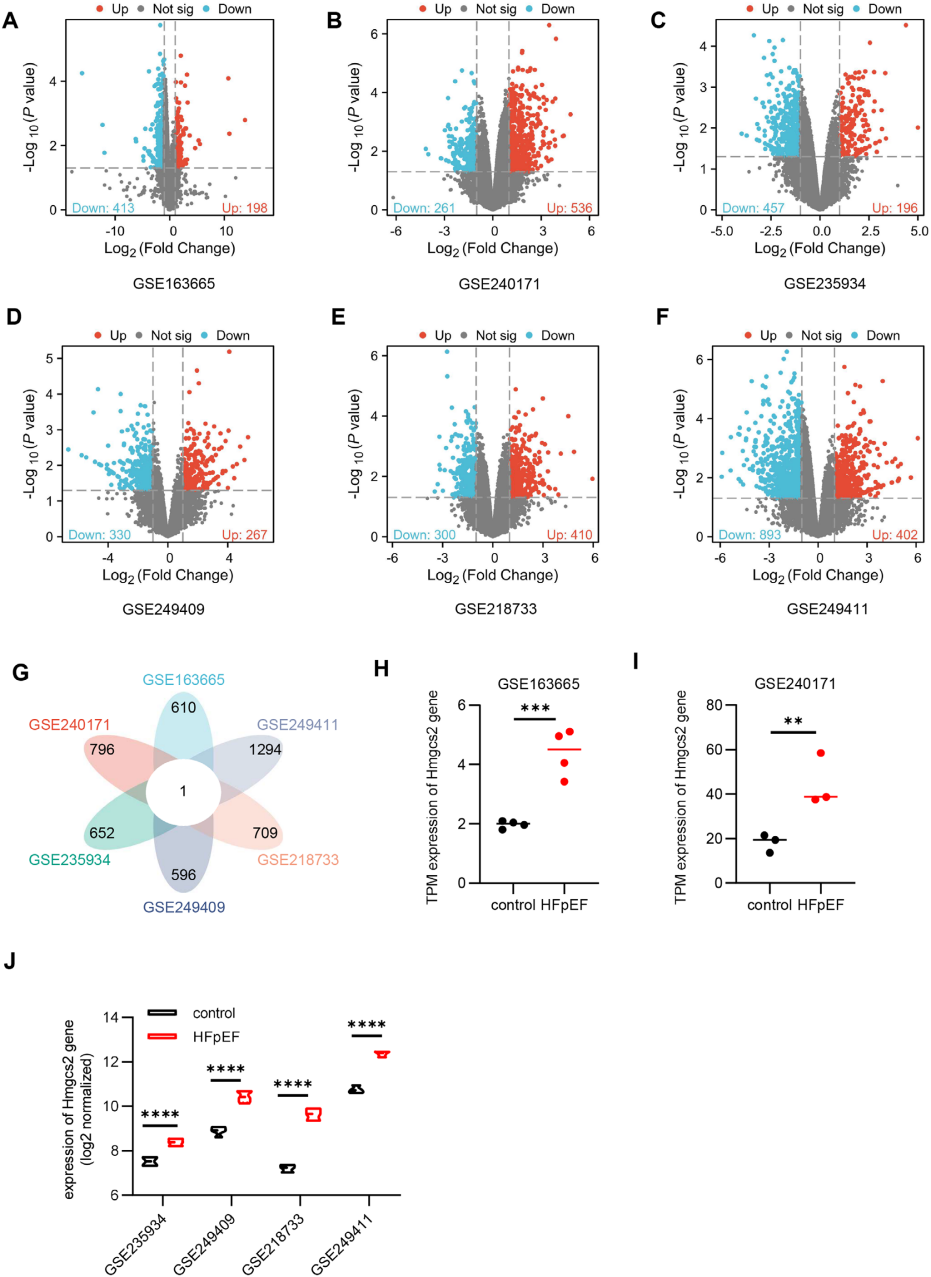
DEGs in metabolic HFpEF. (A–F) Volcano plot of DEGs in GSE163665, GSE240171, GSE235934, GSE249409, GSE218733 and GSE249411 datasets. (G) Petal diagrams showing the number of DEGs that overlap among GSE163665, GSE240171, GSE235934, GSE249409, GSE218733 and GSE249411 datasets. (H) TPM expression of Hmgcs2 gene in GSE163665 dataset. (I) TPM expression of Hmgcs2 gene in GSE240171 dataset. (J) Log2 normalised expression of Hmgcs2 gene in GSE235934, GSE249409, GSE218733 and GSE249411 datasets.

We systematically analysed DEGs in multiple public datasets with metabolic HFpEF to uncover the potential molecular mechanisms. The GSE163665 dataset utilised ZSF1‐obese rats as the HFpEF model, with ZSF1‐lean rats serving as controls. Differential expression analysis identified a total of 611 DEGs, comprising 198 upregulated and 413 downregulated genes (Figure 1A). The GSE240171 dataset employed an HFpEF mouse model induced by AngII combined with HFD, with normally fed mice as controls. This dataset identified 797 DEGs, including 536 upregulated and 261 downregulated genes (Figure 1B). The GSE235934 dataset used leptin receptor‐deficient (db/db) mice as the HFpEF model, with wild‐type mice serving as controls. A total of 653 DEGs were identified, with 196 upregulated and 457 downregulated genes (Figure 1C). The GSE249409 dataset employed an HFpEF mouse model induced by HFD combined with mTAC, with normally fed mice as controls. Analysis revealed 597 DEGs, consisting of 267 upregulated and 330 downregulated genes (Figure 1D). Both the GSE218733 and GSE249411 datasets used an HFpEF mouse model induced by HFD combined with L‐NAME, with normally fed mice as controls. Analysis showed that the GSE218733 dataset (Figure 1E) identified 710 DEGs (410 upregulated and 300 downregulated), while the GSE249411 dataset (Figure 1F) identified 1295 DEGs (402 upregulated and 893 downregulated).

To identify shared molecular features among various metabolic HFpEF animal models, we performed an integrative analysis of DEGs derived from the aforementioned datasets. Petal diagram analysis revealed that, despite heterogeneity in gene expression profiles among different metabolic HFpEF animal models, Hmgcs2 is a commonly upregulated gene in these models (Figure 1H–J).

### 3.2 General Biological and Echocardiography Features of HFpEF Mice

**FIGURE 2:**
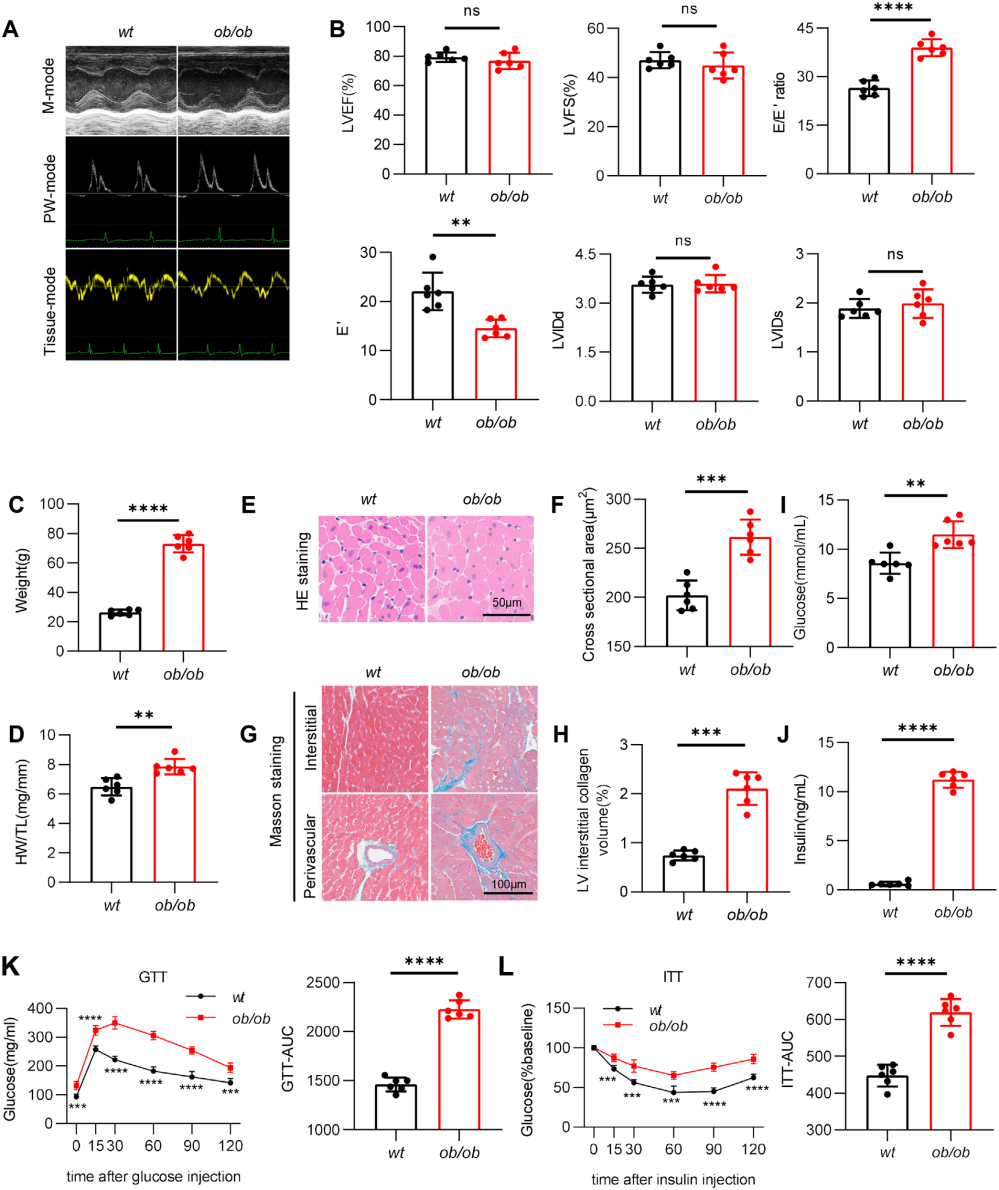
General biological and echocardiography features of HFpEF mice. (A) Representative M‐mode, PW‐mode and Tissue‐mode echocardiographic images of wt and ob/ob mice. (B) Assessments of echocardiographic parameters of left ventricular ejection fraction (LVEF), left ventricular fraction shortening (LVFS), ratio between mitral E wave and E' wave (E/E'), E' wave, left ventricular end‐diastolic internal diameter (LVIDd), and left ventricular end‐systolic internal diameter (LVIDs) in wt and ob/ob mice. (C) Body weight (BW) in wt and ob/ob mice. (D) Heart weight (mg) and tibia length (mm) ratio (HW/TL) in wt and ob/ob mice. (E) Representative images of cardiac haematoxylin–eosin (HE) staining and (F) Quantification of cardiomyocyte cross‐sectional area based on HE staining. (G) Cardiac Masson trichrome staining in the interstitial and peripheral area, and (H) Quantification of the left ventricular interstitial collagen volume. (I) The serum random blood glucose concentration in wt and ob/ob mice. (J) The serum insulin concentration in wt and ob/ob mice. (K) Intraperitoneal glucose tolerance test (ipGTT) (left) and bar graphs depicting the area under the curve of the ipGTT experiment (right). (L) Intraperitoneal insulin tolerance test (ipITT) (left) and bar graphs depicting the area under the curve of the ipITT experiment (right). Data are represented as mean ± SD. *p < 0.05, **p < 0.01, ***p < 0.001, ****p < 0.0001.

Then, we employed ob/ob mice to establish a HFpEF disease model. All mice were housed until 24 weeks and underwent echocardiography at 24 weeks of age. We found no differences in systolic dysfunction between wt and ob/ob mice, as identified by unchanged LVEF and LVFS (Figure 2A,B). However, we discovered that ob/ob mice had significant left ventricle diastolic dysfunction, as significantly increased in early diastolic transmitral flow velocity to early diastolic mitral annular tissue velocity ratio (E/E′) and decreased in early diastolic mitral annular tissue velocity ratio (E′) (Figure 2A,B). Compared to wt mice, ob/ob mice exhibited significant obesity (Figure 2C). Additionally, we assessed cardiac morphology to explore the structural remodelling in the hearts of ob/ob mice. Our findings indicated that myocardial hypertrophy in ob/ob mice, as evidenced by increased heart weight‐to‐tibia length (HW/TL) ratios (Figure 2D). Furthermore, HE staining demonstrated that the cross‐sectional area of the cardiomyocytes in ob/ob mice was significantly larger compared to those from wt mice (Figure 2E,F). Furthermore, ob/ob mice showed obvious cardiac fibrosis, as shown by Masson staining of heart sections (Figure 2G,H).

The ob/ob mice also exhibited systemic dysregulation of glucose metabolism, which was primarily manifested by elevated blood glucose (Figure 2I) and serum insulin levels (Figure 2J), insulin resistance (Figure 2K) and impaired glucose tolerance (Figure 2L). Dyslipidemia was also prominent, with markedly elevated serum levels of triglycerides (TG), total cholesterol (TC), low‐density lipoprotein cholesterol (LDL‐c), high‐density lipoprotein cholesterol (HDL‐c) and non‐esterified fatty acids (NEFA) (Figure S1A–E). Systemic inflammation was indicated by elevated serum C‐reactive protein (CRP) levels (Figure S1F). Additionally, liver dysfunction was observed, as evidenced by increased serum levels of alanine aminotransferase (ALT) and aspartate aminotransferase (AST) (Figure S1G,H). A significant elevation in serum lactate dehydrogenase (LDH) levels (Figure S1I) further confirmed the presence of multi‐organ damage in ob/ob mice. Compared with the control group, ob/ob mice exhibited a significant reduction in running distance, indicating impaired exercise endurance (Figure S1J).

Taken together, these pathological characteristics observed are highly consistent with the clinical manifestations of human HFpEF patients, ensuring the ob/ob mice are an ideal model for investigating metabolic HFpEF.

### 3.3 Proteomic Signature of Heart Tissue in HFpEF Mice

**FIGURE 3:**
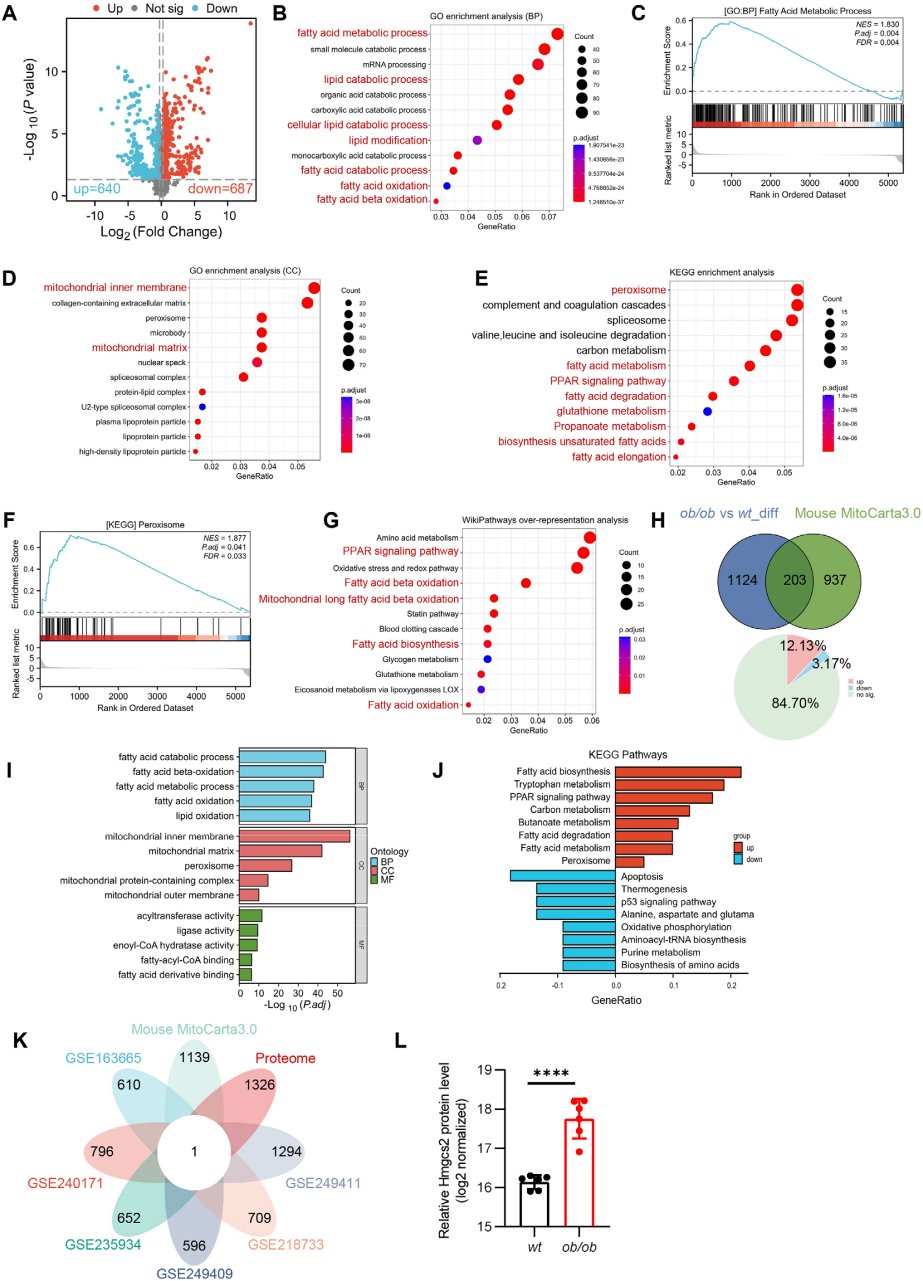
Proteomic signature of heart tissue in HFpEF mice. (A) Volcano plot of DEPs in the heart tissue of ob/ob and wt mice. (B) BP of GO pathway enrichment analysis of DEPs in the heart tissue of ob/ob and wt mice. (C) GSEA analysis of fatty acid metabolic process based on GO analysis of BP. (D) CC of GO pathway enrichment analysis of DEPs in the heart tissue of ob/ob and wt mice. (E) KEGG pathway enrichment analysis of DEPs in the heart tissue of ob/ob and wt mice. (F) GSEA analysis of peroxisome based on GO analysis of CC. (G) Wiki pathway enrichment analysis of DEPs in the heart tissue of ob/ob and wt mice. (H) Venn diagrams (upper) displaying the number of MitoDEPs that overlap between DEPs and MitoCarta3.0. Pie chart (lower) showing the proportion of MitoDEPs in the total DEPs. (I) GO pathway enrichment analysis of MitoDEPs. (J) KEGG pathway enrichment analysis of MitoDEPs. (K) Petal chart displaying the number of differential expressions that overlap between GSE163665, GSE240171, GSE235934, GSE249409, GSE218733, GSE249411, MitoCarta3.0 and Proteome. (L) Log2 normalised Hmgcs2 protein expression level of wt and ob/ob mice in proteome.

Although transcriptomic data from various metabolic HFpEF models have been obtained from the GEO database, these data reflect only changes in gene expression. However, molecular functions are primarily exerted at the protein level. Therefore, obtaining proteomic information from the hearts of HFpEF mice is crucial for comprehensively elucidating the pathological mechanisms of HFpEF. We next isolated the left ventricle of ob/ob mice for proteomic analysis. A total of 1327 DEPs were quantified in ob/ob mice compared with wt mice, among which 640 DEPs were upregulated and 687 DEPs were downregulated (Figure 3A). GO enrichment analysis showed that DEPs were successfully annotated to biological process (BP) functional items, including fatty acid metabolism (Figure 3B). GSEA analysis showed that the fatty acid metabolic process was enriched among up‐regulated genes (Figure 3C). Cellular component (CC) analysis indicated that DEPs were mainly distributed in the mitochondrial inner membrane (Figure 3D). KEGG analysis showed enrichment of pathways mainly involved in peroxisome, fatty acid metabolism and the PPAR signalling pathway (Figure 3E). GSEA analysis showed that the peroxisome was enriched among up‐regulated genes (Figure 3F). Wiki analysis identified functional pathways associated with the PPAR signalling pathway and fatty acid β‐ oxidation (Figure 3G). Concurrently, we observed notable alterations in pathways related to mitochondrial metabolism, and next, we delved deeper into the analysis of DEPs associated with mitochondria. Genes associated with mitochondria were sourced from the MitoCarta3.0 database. An intersection of these mitochondrial‐related genes with the previously identified 1328 DEPs was performed to define a subset known as MitoDEPs. In aggregate, we recognised 203 MitoDEPs (Figure 3H). Upregulated MitoDEPs constitute approximately 12.12% of the total DEPs, and downregulated MitoDEPs account for about 3.16% of the total DEPs (Figure 3H). Subsequently, a functional analysis was carried out to attain a deeper understanding of the biological roles of these MitoDEPs. As evident from the results of GO enrichment analysis and KEGG enrichment analysis, these MitoDEPs were found to be associated with the fatty acid metabolic process (Figure 3I,J).

To further integrate multiple data sources, including transcriptomic datasets, mitochondria‐related differentially expressed genes (MitoDEGs), and differentially expressed proteins identified by proteomics, we aimed to identify consistently dysregulated molecules across different datasets. Ultimately, an overlapping differential expression profile was obtained, in which Hmgcs2 was the only molecule that exhibited a significant difference (Figure 3K). Compared to control samples, proteomic analysis revealed that the expression level of Hmgcs2 in the cardiac tissue of ob/ob mice was approximately 3‐fold higher than that in the control group (Figure 3L).

### 3.4 Runx2 Is the Upstream Transcriptional Regulatory Factor of Hmgcs2

**FIGURE 4:**
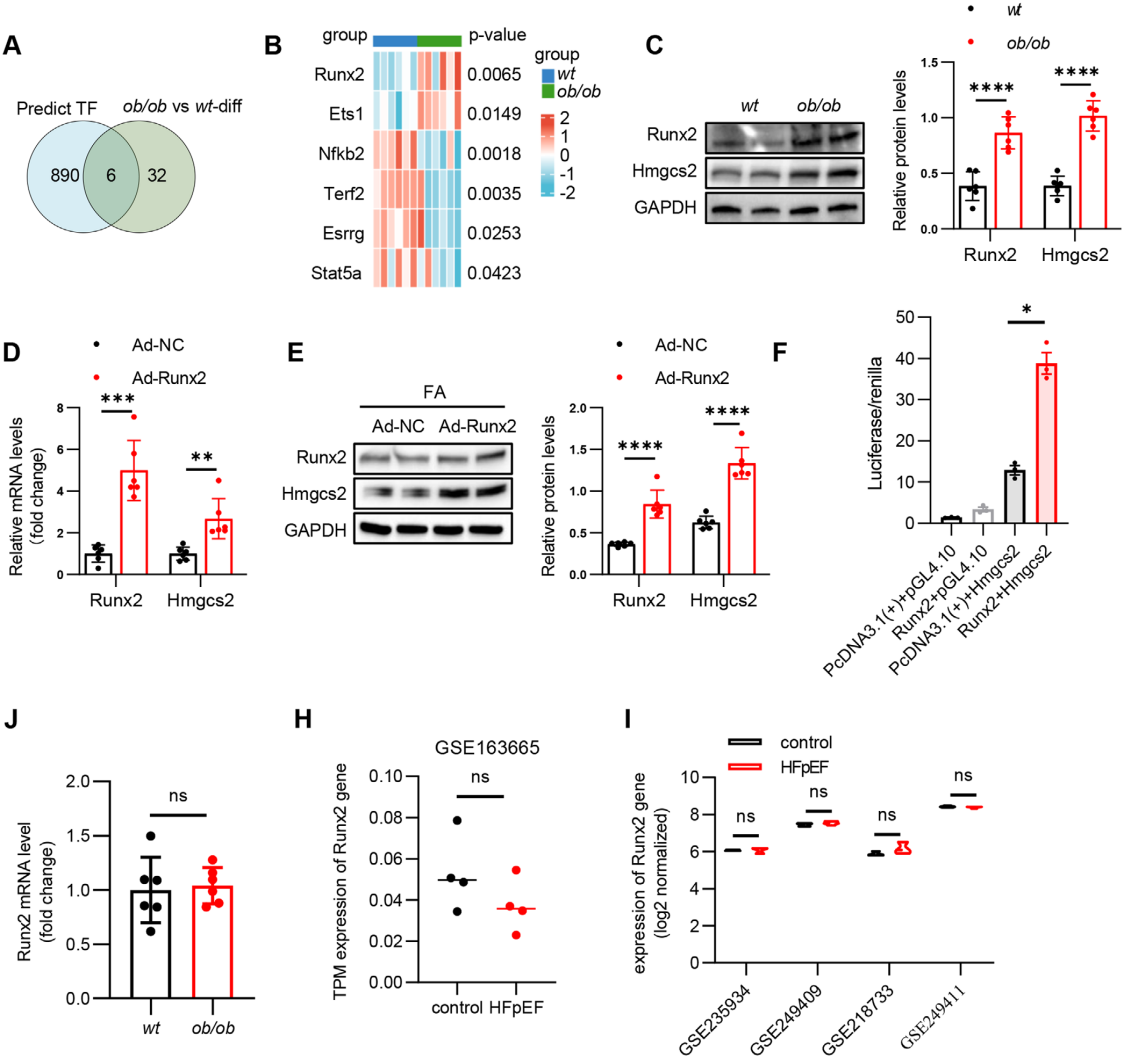
Runx2 is the upstream transcriptional regulatory factor of Hmgcs2. (A) Venn diagrams displaying the number of differentially expressed transcription factors overlapped with predicted transcription factors. (B) Heatmap showing candidates of transcription factors in wt and ob/ob hearts. (C) Representative immunoblotting images (left) showing Runx2 and Hmgcs2 protein levels in heart tissues of wt mice and ob/ob mice; and statistical analyses (right) of relative Runx2 and Hmgcs2 protein levels. (D) Relative mRNA levels of Runx2 and Hmgcs2 in NRVMs infected with Ad‐NC or Ad‐Runx2 followed by stimulation with FA for 24 h. (E) Representative immunoblotting images (left) showing protein levels of Runx2 and Hmgcs2 in NRVMs infected with Ad‐NC or Ad‐Runx2 followed by stimulation with FA for 24 h; and statistical analyses (right) of relative Runx2 and Hmgcs2 protein levels. (F) Luciferase activation driven by the Hmgcs2 promoter after normalisation to renilla luciferase in HEK‐293 cells. (J) Relative mRNA expression of Mib2 genes in wt and ob/ob hearts. (H) TPM expression of Runx2 gene in GSE163665 dataset. (I) Log2 normalised expression of Runx2 gene in GSE235934, GSE249409, GSE218733 and GSE249411 datasets. Data are represented as mean ± SD. *p < 0.05, **p < 0.01, ***p < 0.001, ****p < 0.0001.

Given that both the protein and transcription levels of Hmgcs2 were elevated in HFpEF heart tissue, we hypothesised that Hmgcs2 is primarily regulated at the transcriptional level. AnimalTFDB4.0 was used to predict the transcription factors that could bind to hmgcs2 in mice. These predicted transcription factors, when intersected with those differentially expressed in proteomics, finally obtained 6 transcription factors as intersections (Figure 4A). Notably, among those candidates, Runx2 and Ets1 expression was elevated, while Nfkb2, Terf2, Esrrg and Stat5a expression reduced in the heart tissues of ob/ob mice (Figure 4B). Upregulated Runx2 and Hmgcs2 expression in the hearts of ob/ob mice was confirmed by immunoblotting (Figure 4C). More importantly, in FA‐challenged NRVMs, the mRNA and protein expression of Hmgcs2 was significantly upregulated when Runx2 was overexpressed (Figure 4D,E). A luciferase reporter assay demonstrated that the transfection of plasmids encoding the Hmgcs2 promoter into HEK‐293 cells, which overexpressed Runx2, resulted in an increase in luciferase expression (Figure 4F), highlighting the fact that Runx2 transcriptionally upregulates the expression of Hmgcs2. Next, we further found that the mRNA level of Runx2 remained unchanged in the heart tissues of ob/ob mice (Figure 4J). In the cardiac tissues of other metabolic HFpEF animal models, the transcriptional levels of Runx2 were also unchanged compared with those in the control group (Figure 4H,I). This finding suggests that the elevated protein expression of Runx2 is driven by post‐translational modifications.

### 3.5 Mib2 Promotes the Ubiquitination and Degradation of Runx2

**FIGURE 5:**
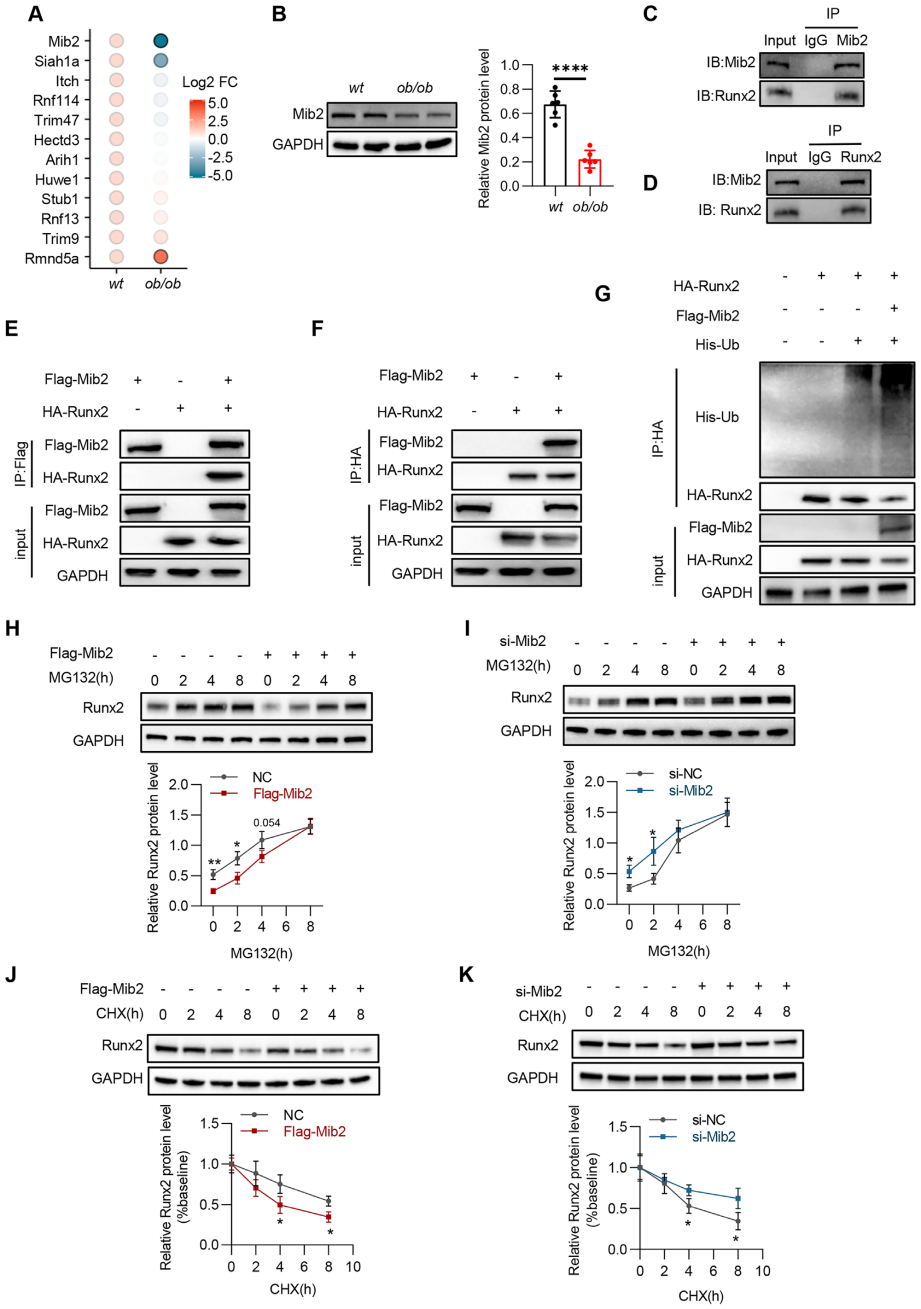
Mib2 promotes the ubiquitination and degradation of Runx2. (A) Dot matrix plot showing differentially expressed E3 Ubiquitin ligases in wt and ob/ob hearts. (B) Representative immunoblotting images (left) showing Mib2 protein levels in heart tissues of wt mice and ob/ob mice and statistical analyses (right) of relative Mib2 protein levels. (C) and (D) Endogenous immunoprecipitation analysis of the interaction of Mib2 and Runx2 in heart tissues of mice using Mib2 or Runx2 antibodies. (E, F) Co‐IP assays of the interaction between Mib2 and Runx2 in HEK293T cells transfected with the indicated plasmids. (G) Ubiquitination assays confirming the ubiquitination of Runx2 after overexpression of Mib2 in HEK293T cells. (H, I) Representative immunoblot image (upper) of Runx2 protein levels in HEK293T cells treated with MG132(10 μmol/L) after transfection with Flag‐Mib2 or si‐Mib2, and quantification of Runx2 level (lower). (J, K) Representative immunoblot image (upper) of Runx2 protein levels in HEK293T cells treated with CHX (10 μmol/L) after transfection with Flag‐Mib2 or si‐Mib2, and quantification of Runx2 level (lower). Data are represented as mean ± SD. *p < 0.05, **p < 0.01, ***p < 0.001, ****p < 0.0001.

We performed an additional analysis targeting DEPs associated with ubiquitinating enzymes to identify E3 ubiquitin ligases that regulate the post‐translational modifications of Runx2. We identified that the protein levels of five E3 ubiquitin ligases were upregulated, whereas those of seven other E3 ubiquitin ligases were downregulated (Figure 5A). Notably, among these, Mib2 exhibited the most significant downregulation in the hearts of ob/ob mice. The protein expression of Mib2 was further confirmed by Western blotting (Figure 5B). We proceeded to evaluate the direct interaction between Mib2 and Runx2 using a co‐immunoprecipitation (Co‐IP) assay. As anticipated, our results confirmed that Mib2 formed a complex with Runx2 in the cardiac tissues of ob/ob mice (Figure 5C,D). The specific interaction between Mib2 and Runx2 was subsequently validated in HEK293T cells that were exogenously infected with Mib2 and Runx2 overexpression plasmid (Figure  5E,F). We then assessed the level of Runx2 ubiquitination through a ubiquitination assay. Consistent with our hypothesis, Mib2 significantly enhanced the ubiquitination of Runx2 in vitro (Figure  5G). Next, we will investigate whether Mib2 promotes Runx2 degradation via the ubiquitin‐proteasome system. We first incubated HEK293T cells with the proteasome inhibitor MG132 and evaluated the Runx2 protein levels. The Runx2 protein accumulation was lower in the Mib2‐overexpressing HEK293T cells after MG132 treatment in comparison with the vector control group (Figure  5H), whereas Runx2 protein accumulation was faster upon MG132 administration in si‐NC cells in comparison with si‐Mib2 cells (Figure  5I). Ultimately, the regulatory influence of Mib2 on Runx2 protein was effectively blocked by MG132 administration (Figure  5H,I). Last, we incubated HEK293T cells with cycloheximide (CHX), an inhibitor of protein translation, and evaluated the Runx2 protein levels. Compared with the control group, Mib2 overexpression significantly shortened the half‐life of Runx2 protein (Figure 5J), whereas Mib2 silencing extended its half‐life (Figure 5K). Taken together, these findings demonstrate that Mib2 specifically binds to Runx2, facilitating its ubiquitination and subsequent degradation.

### 3.6 Mib2 Regulates Lipid Accumulation in a Hmgcs2‐Dependent Manner

**FIGURE 6:**
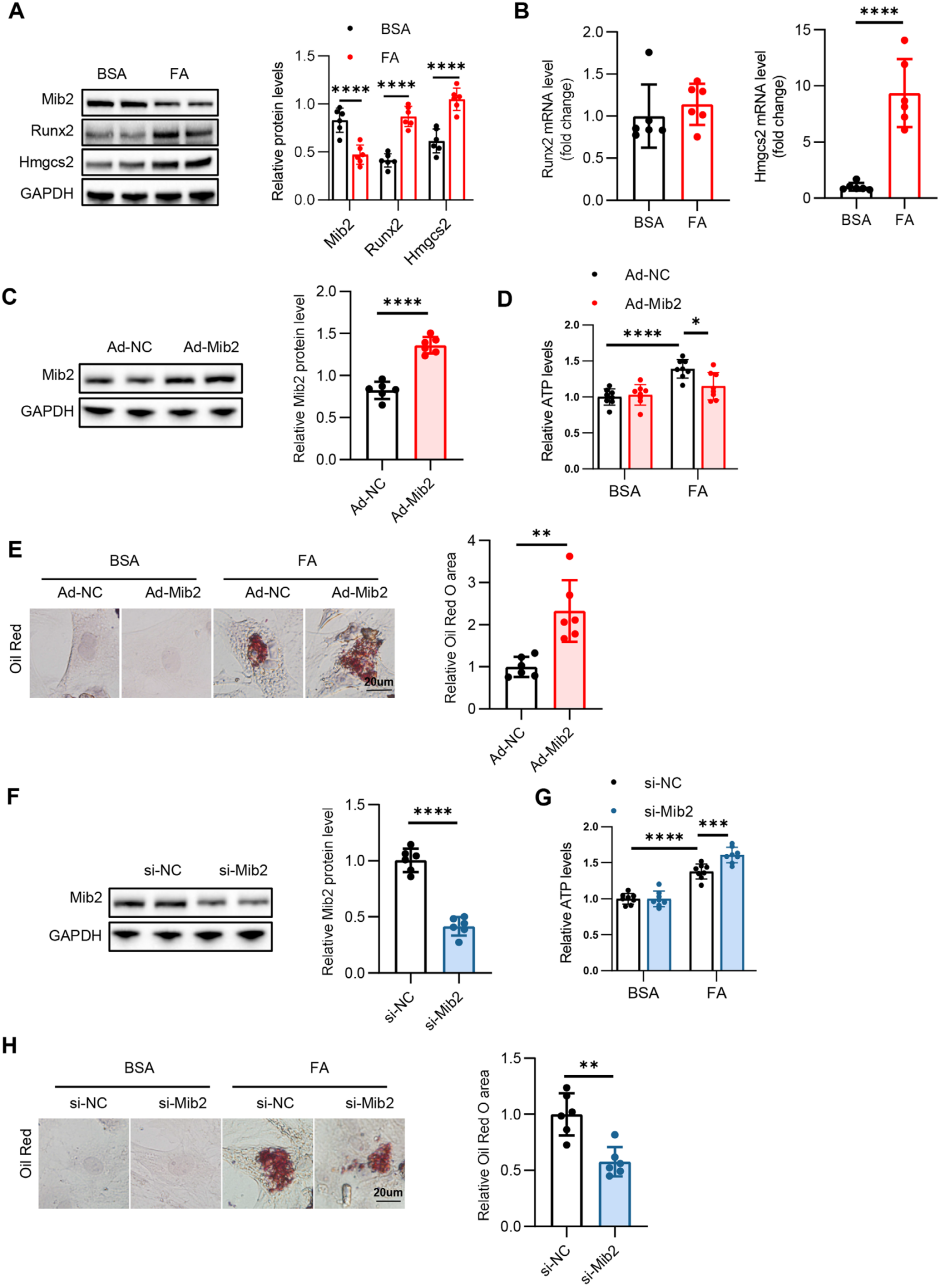
Mib2 regulates lipid accumulation in a Hmgcs2‐dependent manner. (A) Representative immunoblotting images (left) showing protein levels in NRVMs treated with FA and statistical analyses (right) of relative protein levels. (B) Relative mRNA expression of Runx2 and Hmgcs2 genes in NRVMs treated with FA. (C) Representative immunoblotting images (left) of Mib2 protein levels in NRVMs after transfection with Ad‐Mib2 and statistical analyses (right) of relative Mib2 protein levels. (D) Relative ATP levels in NRVMs after transfection with Ad‐Mib2. (E) Representative Oil Red O staining (left) in NRVMs after transfection with Ad‐Mib2 and quantitative analyses (right) of Oil Red O staining. (F) Representative immunoblotting images (left) of Mib2 protein levels in NRVMs after transfection with si‐Mib2 and statistical analyses (right) of relative Mib2 protein levels. (G) Relative ATP levels in NRVMs after transfection with si‐Mib2. (H) Representative Oil Red O staining (left) in NRVMs after transfection with si‐Mib2 and quantitative analyses (right) of Oil Red O staining. Data are represented as mean ± SD. *p < 0.05, **p < 0.01, ***p < 0.001, ****p < 0.0001.

We next treated NRVMs with FA (palmitate (PA): oleate (OA) = 1:1) for 24 h to mimic hyperlipidemia in vivo. In line with our findings in ob/ob mice, Mib2 protein expression was markedly decreased and Rux2 and Hmgcs2 protein expression was markedly increased in the FA‐treated NRVMs (Figure 6A). The mRNA expression level of Hmgcs2 is increased, while the mRNA expression level of Runx2 remains unchanged (Figure 6B). To evaluate the function of Mib2 in lipid metabolism in vitro, we incubated NRVMs with FA medium with Ad‐Mib2, and immunoblotting analysis confirmed the successful overexpression of Mib2 in NRVMs (Figure 6C). We first measured the ATP production and discovered that the FA‐induced increase in ATP was attenuated by Mib2 overexpression (Figure 6D). Additionally, Mib2 overexpression exacerbated lipid accumulation in FA‐treated NRVMs (Figure 6E). Then, we incubated NRVMs with FA medium along with si‐Mib2, and immunoblotting confirmed the efficient knockdown of Mib2 in NRVMs upon si‐Mib2 transfection (Figure 6F). Mib2 silence increased ATP production (Figure 6G) stimulated by FA and reduced lipid storage in FA‐incubated NRVMs, as indicated by a diminished Oil Red O staining area within the cells (Figure 6H). Next, we investigated whether Hmgcs2 is essential for the effect of Mib2 on lipid metabolism. We used si‐Hmgcs2 to suppress Hmgcs2 expression in FA‐stimulated NRVMs (Figure S2A). Notably, knockdown of Hmgcs2 abolished the promoting effect of Mib2 silence on ATP production and its inhibitory role in the accumulation of lipids (Figure S2B,C). Taken together, these data suggest that Hmgcs2 serves as an essential target of Mib2 in lipid metabolism and that targeting the Mib2‐hmgcs2 axis may provide therapeutic strategies for lipid metabolism.

### 3.7 Cardiac‐Specific Mib2 Overexpression Aggravates Diastolic Dysfunction and Lipid Accumulation

**FIGURE 7:**
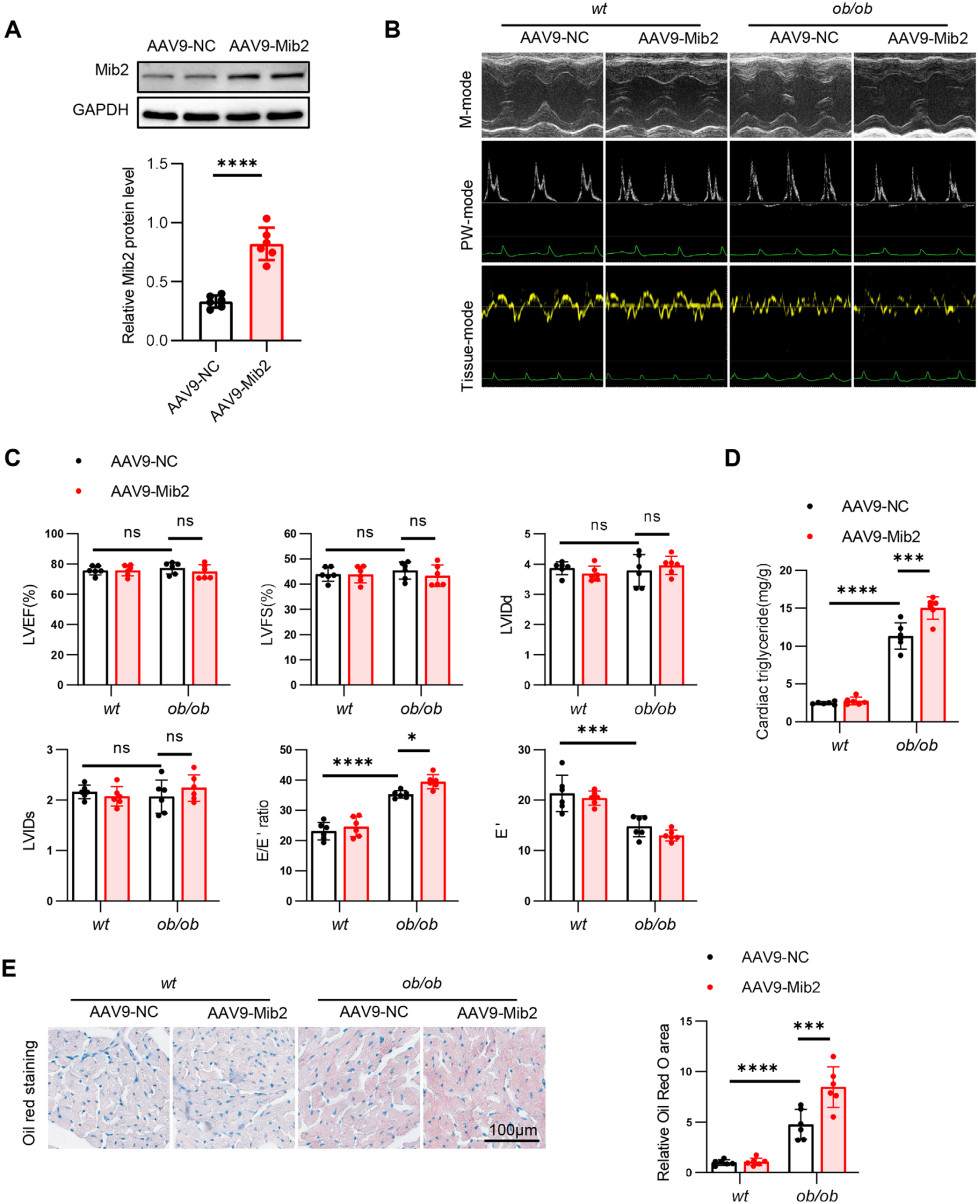
Cardiac‐specific Mib2 overexpression aggravates diastolic dysfunction and lipid accumulation. (A) Representative immunoblotting images (left) of Mib2 protein levels in heart tissues after infection with AAV9‐Mib2 and statistical analyses (right) of relative Mib2 protein levels. (B) Representative M‐mode, PW‐mode and Tissue‐mode echocardiographic images of the left ventricle after infection with AAV9‐Mib2. (C) Assessments of echocardiographic parameters of left ventricular ejection fraction (LVEF), left ventricular fraction shortening (LVFS), ratio between mitral E wave and E' wave (E/E'), E' wave, left ventricular end‐diastolic internal diameter (LVIDd), and left ventricular end‐systolic internal diameter (LVIDs). (D) Cardiac triglyceride content in myocardial tissue from wt and ob/ob mice after infection with AAV9‐Mib2. (E) Representative Oil Red O staining (left) images in heart tissue from wt and ob/ob mice after infection with AAV9‐Mib2 and quantitative analyses (right) of Oil Red O staining. Data are represented as mean ± SD. *p < 0.05, **p < 0.01, ***p < 0.001, ****p < 0.0001.

To elucidate the role of Mib2 in HFpEF, we specifically overexpressed Mib2 in the heart by administering AAV9‐cTnT‐Mib2 via tail vein injection into wt and ob/ob mice. The efficiency of Mib2 overexpression in myocardial tissues was verified using immunoblotting (Figure 7A). Echocardiographic evidence revealed that ob/ob mice presented clear hallmarks of diastolic dysfunction, including a marked increase in E/E' ratio and decreased in E' (Figure 7B,C). AAV9‐Mib2 further exacerbated cardiac diastolic function, as evidenced by an increase in the E/E' ratio, although E' only showed a trend to decrease (Figure 7B,C). We subsequently measured the cardiac triglyceride content and found that there was a significant increase in lipid accumulation in the hearts of mice with cardiomyocyte‐specific overexpression of Mib2 (Figure 7D). The Oil Red O staining results also showed more pronounced lipid accumulation in the heart tissues of Mib2‐overexpressing mice (Figure 7E).

## 4 Discussion

The global spread of obesity and metabolic disorders is reshaping the landscape of cardiovascular disease, a trend that is anticipated to accelerate [15, 16]. A significant aspect of this phenomenon is the rise in prevalence of HFpEF, particularly cardiometabolic HFpEF. Abnormal activation of lipid metabolism in the heart was observed in ob/ob mice exhibiting the HFpEF phenotype. Subsequently, this study demonstrated that downregulated Mib2 expression in the heart of HFpEF regulated the lipid metabolism. Mechanically, Mib2 promotes the ubiquitination of Runx2 to degrade Runx2, therefore inhibiting Hmgcs2 transcription and lipid homeostasis, ultimately enhancing cardiac dysfunction in HFpEF. This hypothesis is supported by our results that restoration of Mib2 exacerbated cardiac dysfunction and lipid storage in the heart, which was further verified by NRVMs that Mib2 overexpression exacerbates cardiomyocyte steatosis in vitro assay.

### 4.1 Hmgcs2 Is Upregulated in Various Animal Cardiometabolic HFpEF Models

HFpEF is characterised as a multifactorial disease. In addition to typical clinical signs and symptoms of HF, non‐cardiac comorbidities frequently co‐exist and contribute to the pathophysiology of HFpEF [17]. In the past several decades, a multitude of small animal models have been developed to replicate the diverse pathophysiological mechanisms that contribute to the development of HFpEF [18, 19]. Considering the intricate pathophysiology of HFpEF and the heterogeneity of clinical phenotypes in humans, we attempted to identify commonalities among different preclinical HFpEF models. Therefore, we downloaded the transcriptome data of heart tissues in cardiometabolic HFpEF from the GEO database, and these HFpEF models were generated using five different methodologies. By analysing six different datasets, we particularly noted significant changes in the metabolic‐related gene Hmgcs2. The Hmgcs2 protein is localised to the mitochondria, serving as a rate‐limiting enzyme for catalysing the first reaction of ketogenesis, where it catalyses the fate‐committing condensation of fatty acid β‐oxidation‐derived acetoacetyl‐CoA (AcAc‐CoA) and acetyl‐CoA to generate HMG‐CoA [20]. Although primarily expressed in the liver under normal physiological conditions [21], the potential role of Hmgcs2 in cardiovascular diseases has been gradually revealed in recent years. Cardiomyocyte‐specific Hmgcs2 knockout mice showed worsened heart function following cardiac ischaemia reperfusion injury, which can be rescued by the induction of exogenous Hmgcs2 by intramyocardial AAV9 injection [22].

While heterogeneity is frequently considered a significant barrier in preclinical heart HFpEF research, we contest this view and posit that Hmgcs2 is upregulated in different HFpEF animal models utilising a combined transcriptomics and proteomics analysis. It implies that the expression levels of Hmgcs2 could be significantly altered in response to pathological alterations in the heart, potentially playing a pivotal role in the processes.

### 4.2 The Ob/Ob Mouse Is an HFpEF Model Accompanying Metabolic Syndrome

The development of efficacious treatments is fundamentally dependent on solid preclinical models that accurately reflect the essential aspects of the clinical condition and also allow for thorough investigation of potential disease mechanisms within the scope of phenotypes that are relevant to clinical practice. The ob/ob mouse model is characterised by a deficiency in leptin, leading to the spontaneous development of obesity and type 2 diabetes mellitus secondary to hyperglycemia and hyperinsulinemia [23, 24]. The mice exhibit concentric cardiac hypertrophy and may experience diastolic dysfunction, potentially attributable to the accumulation of lipids [25]. Therefore, we used ob/ob mice to establish a HFpEF disease model, which can more comprehensively reflect both cardiac and extra‐cardiac manifestations. These pathological characteristics observed in ob/ob mice are highly consistent with the clinical manifestations of human HFpEF patients, ensuring the ob/ob mice as an ideal model for investigating metabolic HFpEF.

### 4.3 Runx2 Is the Upstream Transcriptional Regulatory Factor of Hmgcs2

Runx2 is a key osteogenic transcription factor that plays a crucial role in bone development [26]. In recent years, its role in cardiovascular diseases has attracted increasing attention, particularly in vascular calcification and cardiac remodelling. Under normal conditions, Runx2 expression is low in the vascular system. However, it is significantly upregulated in the calcified arteries of humans and animal models in atherosclerosis, diabetes and chronic kidney disease [27, 28, 29, 30]. In vitro studies have shown that Runx2 upregulation promotes calcification in various vascular cells, including vascular smooth muscle cells, endothelial cells and vascular progenitor cells [27, 28, 29, 30]. Additionally, Runx2 regulates the endothelial‐mesenchymal transition (EndMT) of cardiac microvascular endothelial cells [31], a process that plays a key role in cardiac fibrosis and disease progression. In pathological cardiac hypertrophy, the liquid–liquid phase separation (LLPS) of Runx2 has been identified as a regulated form underlying ventricular remodelling. As a key transcription factor, Runx2 dynamically regulates the activity of cellular signalling networks via LLPS, driving the hypertrophic response of cardiomyocytes by activating the epidermal growth factor receptor (EGFR) signalling pathways [32].

In this study, a significant elevation in the protein level of Runx2 was observed in the cardiac tissues of ob/ob mice. Dual‐luciferase reporter gene assays confirmed that Runx2 directly binds to the promoter region of Hmgcs2 and significantly enhances its transcriptional activity. The innovative findings reveal a novel function of Runx2 in the cardiovascular system. This discovery not only enhances our understanding of the biological functions of Runx2 but also provides a new potential therapeutic target for cardiometabolic HFpEF.

### 4.4 E3 Ligase Mib2 Regulates the Ubiquitination and Degradation of Runx2

The process of ubiquitination, which involves multiple steps, is regulated by the coordinated action of E1, E2 and E3 enzymes that sequentially activate, conjugate and ultimately ligate ubiquitin to the target protein substrates. Recent bioinformatics analyses indicate that humans express a total of 634 E3 ubiquitin ligases [33], which are essential components of the ubiquitin‐proteasome‐mediated protein degradation system, playing a critical role in various areas of cell biology [34, 35]. Among which, the biological function of Mib2 was reported primarily in cancer research. It controls Yap/Taz protein degradation to inhibit angiogenesis in tumors [36], and the ubiquitination of PD‐L1 by Mib2 is required for tumour cell immune evasion [37]. In terms of metabolisms, Mib2 promoted PTEN proteasomal degradation to inhibit FoxO1‐dependent Ucp1 (Uncoupling protein‐1) transcription in brown adipose tissue, which triggers obesity [38]. Our study provided the first evidence that Mib2 could significantly regulate the ubiquitination and degradation of Runx2, a critical transcription factor in vascular calcification [39, 40], valve calcification [41, 42] and pathological cardiac hypertrophy [32]. This discovery not only broadens the understanding of the biological functions of Mib2 but also provides a new potential therapeutic target for cardiovascular diseases.

### 4.5 Improving Lipid Overload in Heart Can Reverse HFpEF Phenotypes

It is crucial to recognise that the aetiology of HFpEF has been demonstrated to have a significant association with systemic metabolic dysregulations [43]. Indeed, metabolic disorder‐related HFpEF is the predominant phenotype observed in community settings [1, 44], in which we are particularly concerned about lipid metabolic abnormalities [45]. Previous research has detailed fatty infiltration of cardiomyocytes to trigger cardiac lipotoxicity in failing hearts, causally linking cardiac lipid overload with diastolic dysfunction [46, 47]. In a study, there was an observed buildup of epicardial adipose tissue (EAT), which was found to be linked to adverse hemodynamic and metabolic profiles, along with decreased survival rates among individuals suffering from HFpEF [48]. Parallelly, EAT density was an independent impact factor of cardiometabolic risk in HFpEF [49]. Experimental studies have found that reducing cardiac lipid accumulation can improve the HFpEF phenotype in mice [14]. Recent clinical trial results have shown that in obese patients with HFpEF and type 2 diabetes, 1 year of treatment with semaglutide, which has been employed for the treatment of obesity, overweight individuals and type 2 diabetes [50, 51], effectively improved heart failure‐related symptoms and physical limitations, and reduced bodyweight [52]. Our study shows that Mib2 regulates lipid metabolism in HFpEF heart through the Runx2‐Hmgcs2 axis. Hmgcs2 protein catalyses the fate committing condensation of fatty acid β‐oxidation‐derived AcAc‐CoA and acetyl‐CoA to generate HMG‐CoA [20]. As a downstream of fatty acid β‐oxidation, Hmgcs2 is capable of promoting the degradation of fatty acid [53]. We believe that the increase in Hmgcs2 in heart tissue from HFpEF represents an adaptive response and metabolic reprogramming [54] that helps the failing heart to accelerate lipid metabolism and resist the accumulation of lipids in the heart.

### 4.6 Study Limitations

Nonetheless, there are some limitations in this study. First, our research is based on a publicly accessible dataset, and the outcomes we have derived are predominantly exploratory in nature, potentially deviating to some extent from the factual outcomes. Second, we did not validate the effect of Mib2 knockout on HFpEF at the in vivo level, nor did we explore the role of the RunX2‐Hmgcs2 axis in HFpEF mice. More disappointingly, we did not acquire and analyse a human transcriptome dataset, and we were also constrained from obtaining cardiac tissues from patients for additional validation due to ethical restrictions.

## 5 Conclusion

In summary, we found the significant upregulation in the metabolic‐related gene Hmgcs2 by comprehensive bioinformatics analysis. We have discovered a biological axis, Mib2‐Runx2‐Hmgcs2, in the regulation of cardiac steatosis in HFpEF, providing a promising therapeutic strategy for cardiometabolic HFpEF.

## Author Contributions

**Zhulan Cai:** data curation (lead), formal analysis (lead), methodology (lead), visualization (lead), writing – original draft (lead). **Xiaohua Xiao:** funding acquisition (supporting), resources (supporting), writing – review and editing (equal). **Shunyao Xu:** funding acquisition (equal), project administration (supporting), resources (supporting). **Chen Liu:** conceptualization (equal), funding acquisition (lead), investigation (equal), resources (supporting), validation (equal). **Lingyun Zu:** conceptualization (equal), investigation (equal), supervision (equal), visualization (equal), writing – review and editing (equal).

## Ethics Statement

The study's animal experiments were granted approval by the Medical Ethics Committee of the First Affiliated Hospital of Shenzhen University, with the ethics approval number being 202300207.

## Consent

The authors have nothing to report.

## Conflicts of Interest

The authors declare no conflicts of interest.

## Supporting information


Figures S1–S2.

## Data Availability

The datasets analysed in this study are accessible through the GEO database (https://www.ncbi.nlm.nih.gov/geo/) and MitoCarta3.0 (http://www. broadinstitute.org/mitocarta).
