# Supplementary material for: Mib2 Regulates Lipid Metabolism in Heart Failure With Preserved Ejection Fraction via the Runx2–Hmgcs2 Axis
Source: J Cell Mol Med. 2025 Mar 30;29(7):e70514. doi: 10.1111/jcmm.70514 (PMC11955417; doi:10.1111/jcmm.70514)
Supplement: Supplementary file 1 — Figures S1–S2. [file JCMM-29-e70514-s001.docx]

**
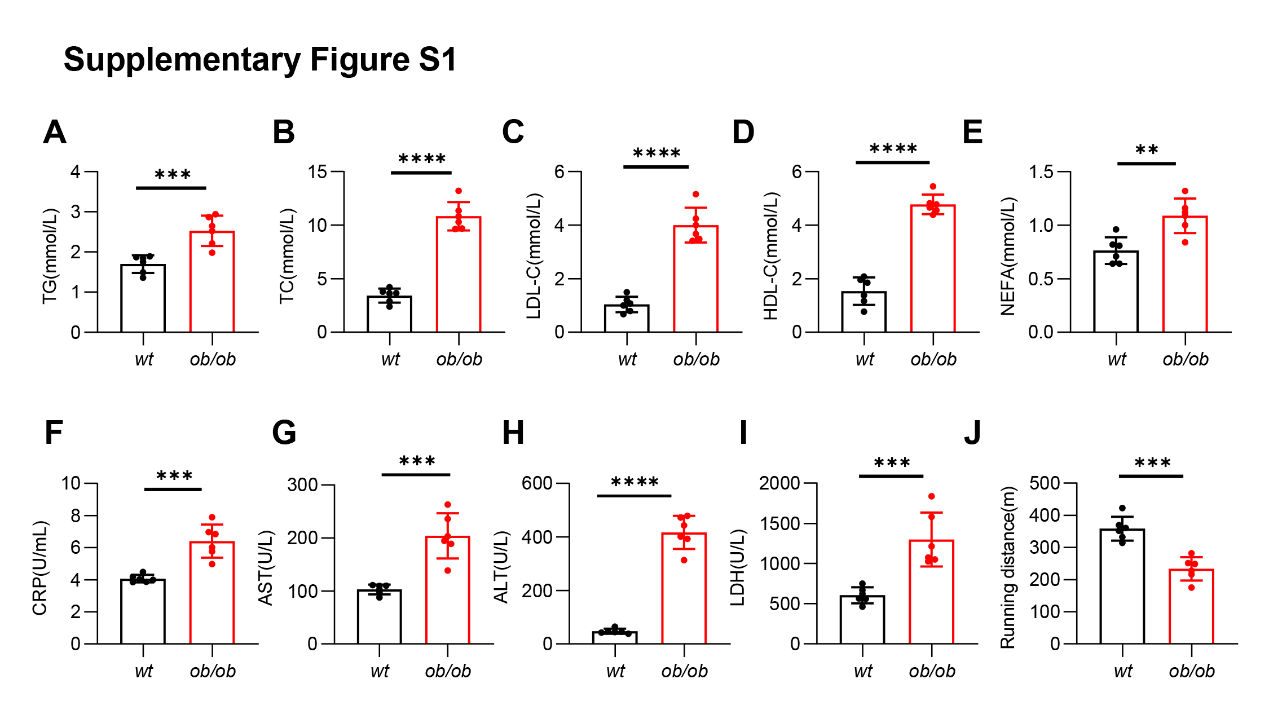
**

**Supplementary Figure S1**

**A:** The serum TG concentration in wt and ob/ob mice. **B:** The serum TC concentration in wt and ob/ob mice. **C:** The serum LDL-C concentration in wt and ob/ob mice. **D:** The serum HDL-C concentration in wt and ob/ob mice. **E:** The serum NEEA concentration in wt and ob/ob mice. **F:** The serum CRP concentration in wt and ob/ob mice. **G:** The serum AST concentration in wt and ob/ob mice. **H:** The serum ALT concentration in wt and ob/ob mice. **I:** The serum LDH concentration in wt and ob/ob mice. **J:** Running distance during exercise exhaustion test in wt and ob/ob mice.

Data are represented as mean±SD. **p*<0.05, ***p*<0.01, ****p*<0.001, *****p*<0.0001.


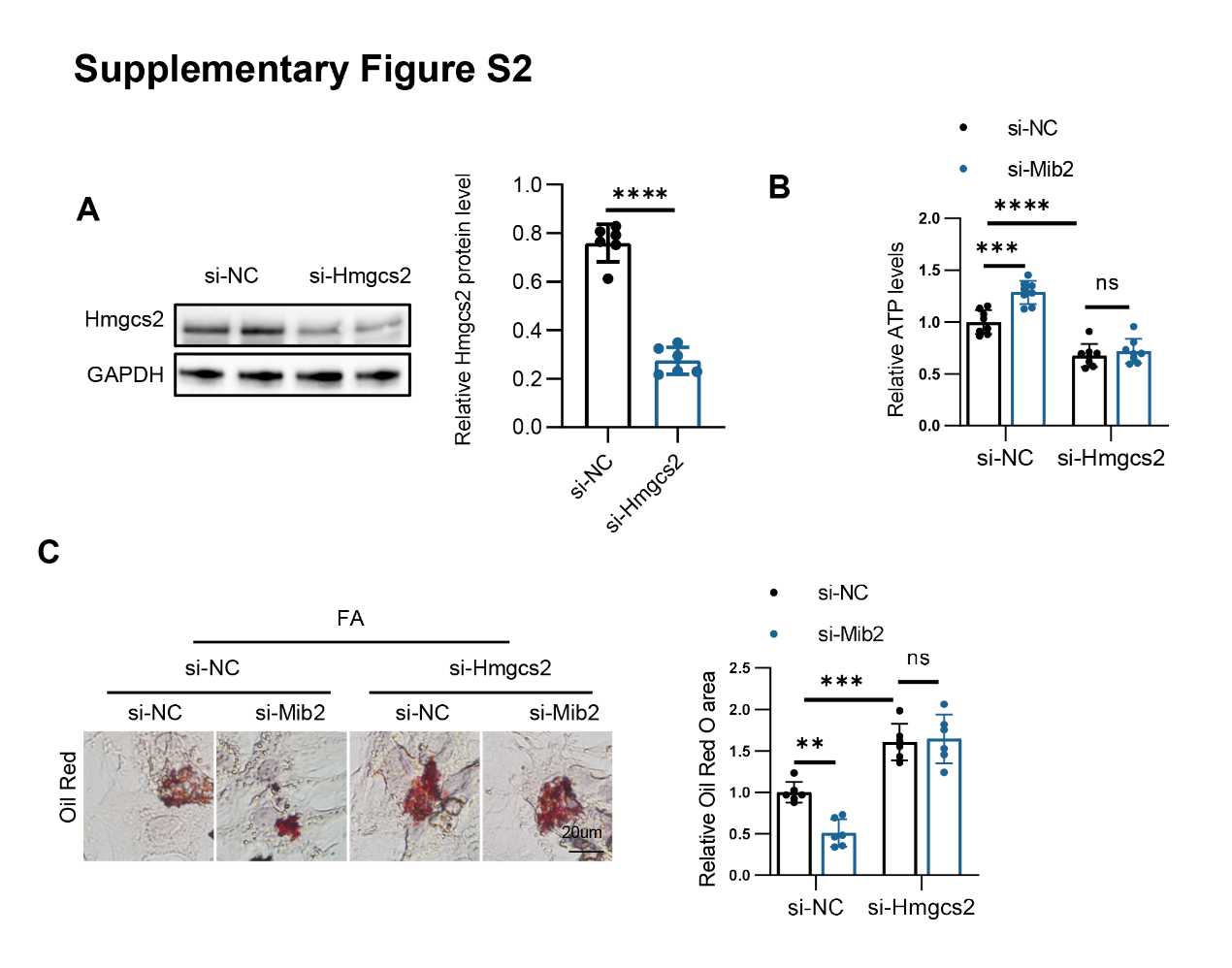


**Supplementary Figure S2**

**A:** Representative immunoblotting images (left) of Hmgcs2 protein levels in NRVMs after transfection with si-Hmgcs2 and statistical analyses (right) of relative Hmgcs2 protein levels. **B:** Relative ATP levels in NRVMs after transfection with si-Hmgcs2 and si-Mib2. **C:** Representative Oil Red O staining (left) in NRVMs after transfection with si-Hmgcs2 and si-Mib2 and quantitative analyses (right) of Oil Red O staining.

Data are represented as mean±SD. **p*<0.05, ***p*<0.01, ****p*<0.001, *****p*<0.0001.
